# Sotos syndrome: An interesting disorder with gigantism

**DOI:** 10.4103/0972-2327.42941

**Published:** 2008

**Authors:** A. Nalini, Arundhati Biswas

**Affiliations:** Department of Neurology, National Institute of Mental Health and Neurosciences, Bangalore, India; 1 Department of Neurosurgery, National Institute of Mental Health and Neurosciences, Bangalore, India

**Keywords:** Cerebral gigantism, epilepsy, primary optic atrophy, sotos syndrome

## Abstract

We report the case of a 16-year-old boy diagnosed to have Sotos syndrome, with rare association of bilateral primary optic atrophy and epilepsy. He presented with accelerated linear growth, facial gestalt, distinctive facial features, seizures and progressive diminution of vision in both eyes. He had features of gigantism from early childhood. An MRI showed that brain and endocrine functions were normal. This case is of interest, as we have to be aware of this not so rare disorder. In addition to the classic features, there were two unusual associations with Sotos syndrome in the patient.

## Introduction

Sotos syndrome, or cerebral gigantism first described by Sotos in 1964, is a syndrome of accelerated linear growth during early childhood and is associated with craniofacial and physical abnormalities.[1] The cause of Sotos syndrome remains unknown, but, most often, it occurs sporadically, though occasional cases with autosomal dominant inheritance have been reported.[2] The clinical features include mental retardation with facial dysmorphic features of frontal bossing, hypertelorism, macrocrania, prognathism, high arched palate and large hands and feet.[1] Excessive prenatal and postnatal overgrowth in height, weight and bone age are present. It is a nonhormone mediated accelerated growth disorder, which mimics pituitary gigantism, but with no neuroendocrine dysfunction.[3] The mandatory criteria for Sotos syndrome include presence of macrocephaly and facial gestalt; the minor criteria are overgrowth and advanced bone age.[4] Cole *et al.* have proposed a diagnostic criteria which includes facial gestalt, growth pattern, bone age and developmental delay.[5] Various rare associations have been reported in Western literature and these include unilateral glaucoma,[6] optic disc pallor and retinal atrophy,[7] gastric carcinoma,[8] septo-optic dysplasia,[9] epilepsy[10] and congenital cardiopathy.[11] The disease is said to be not uncommon and can be missed easily in a clinical setting, if the clinician is not vigilant. Thus, Sotos syndrome is not a rare disorder and can be diagnosed among children with global developmental delay and macrocephaly.[12]

## Case Report

In 2001, a 16-year-old boy presented seizures of six years duration. It was of primary generalized tonic clonic type. He had one to two episodes in a fortnight. He was started on phenytoin and became seizure free. From age 12 onwards, he developed insidious onset and progressive bilateral diminution of vision, without field defects. He was born of a normal delivery, after prolonged labor, and was noted to be a big baby. There was presence of umbilical hernia at birth and he was operated on the second day of life. Macroglossia and prognathism were noted since birth. Neonatal period was uneventful. He had delayed developmental milestones with poor scholastic performance. At three years, he was noticed to have frontal bossing and big hands and feet; at nine years of age, he had a growth spurt; and, by 13 years of age, he attained a height of 185 cm (father aged 62 years was 165 cm tall and mother aged 50 years was 155 cm tall). There was history of excessive somnolence, with lack of interest in performing any work. At seven years of age, he was operated for cryptorchidism. There was no family history of consanguinity, mental deficiency, gigantism or neurofibromatoses.

During the first visit, the patient was evaluated by neurosurgeons for acromegaly, with contrast MRI of the brain, which was normal.

**Figure 1:**
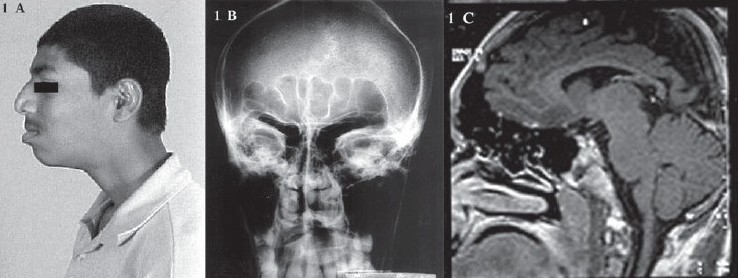
(A) Photograph showing prominent superciliary arches, big nose and prognathism (B) Radiograph of skull showing prominent frontal and maxillary sinuses (C) MRI showing normal brain parenchyma and large sphenoid, frontal and ethmoidal sinuses with extensive pneumatization

Examination revealed a tall boy with occipitofrontal head circumference of 59 cms, (> 98^th^ percentile), height of 185 cms (above 95^th^ centile as per the National Centre for Health Statistics Standards) and weight of 62 kgs, which was appropriate for the age and height (75^th^ centile). He had hypertelorism, megalophthalmos, macroglossia, prognathism, malocclusion of teeth, big nose [Figure 1A], prominent superciliary arches, divergent squint, high arched palate and large hands and feet. There was no scoliosis. Fundi revealed bilateral optic atrophy with visual acuity of 2/60 in right eye and 6/60 in left eye, with total color blindness. The macula was normal and no retinal pigmentary changes were noted. The person had moderate myopia on the right eye and simple myopic astigmatism in the left eye. Anti mongoloid slant of left palpebral aperture was seen. Ocular tension was 14.6 mmHg in both eyes, palpebral aperture of 30 mm in both eyes, intermedial canthal distance of 30 mm and interpupillary distance of 65 mm. Hypertelorism was apparent due to the divergent squint. Exophthalmometry showed 20 mm on both sides. He had mental subnormality; his speech was normal. Binet-Kamath intelligence test was done, which showed evidence of moderate mental retardation with an IQ of 34. There were no psychiatric manifestations. Motor system revealed normal tone and power. The deep tendon reflexes were normal. Sensory and cerebellar systems were normal.

Further investigations revealed normal hormone concentrations in plasma, including plasma growth hormone levels. Analysis for fragile X syndrome was negative. Blood fasting glucose was 94 mg/dl, calcium was 10.5 mg/dl and phosphorus was 4.6 mg/dl. Liver, renal and thyroid functions were normal. EEG showed evidence of recurrent generalized sharp wave discharges. Radiographs of skull showed prominent jaw bone, prominent frontal and maxillary sinuses [Figure 1B]. Radiographs, based on the epiphyseal centres of the hands and feet, did not show evidence of advanced bone age. There was no evidence of campodactyly or widened distal bones. An MRI of the brain was normal, except for large sphenoid, frontal and ethmoidal sinuses, with extensive pneumatization [Figure 1C]. 2D-echocardiogram revealed floppy mitral valve with mild prolapse and Doppler study showed trivial mitral regurgitation.

## Discussion

Sotos syndrome is characterized by growth and development anomaly, with unusual growth spurt commencing around birth which later plateaus by four to five years of age. They have features of gigantism, with nonprogressive neurologic disorder and normal endocrine function. The pathophysiology is not well understood, although it is attributed to disorder of collagen and elastin synthesis, with a common underlying basis between various syndromes like Beckwith-Wiederman, Klippel Feil Trenauney and Weavers syndrome.[13]

Reports confirm that NSD1 mutations are the major cause of Sotos syndrome.[14,15] Weaver's syndrome is similar and closely related to Sotos syndrome, with advanced carpal maturation, widened distal long bones and campodactyly. NSD1 intragenic mutation is also noted in Weaver's patients.[4] Our patient did not have any of these bone abnormalities.

Various MRI abnormalities have been described, particularly presence of large ventricles, midline anomalies including agenesis or hypoplasia of corpus callosum, cavum septum pellucidum and cavum vergae. The rarer findings are heterotopias and macrocerebellum.[5,16,17] Thus, several of these neuroimaging abnormalities provide support for the hypothesis of delayed or disturbed development of the brain.[5] However, our patient showed no brain abnormality, suggesting that these may be absent in Sotos syndrome.

Several reports of classical Sotos syndrome have been reported in Western literature. Its association with seizures and primary optic atrophy is rare. Though subclinical EEG abnormalities are known, clinical seizures are very uncommon.[10] In a report from Saudi Arabia, 43% of 14 patients with Sotos syndrome had epilepsy.[18] West syndrome has also been reported in a patient with Sotos syndrome.[19] In a study performed on a large population from several colleges and schools of optometry and various clinics of optometrists and ophthalmologists, it was found that moderate to high refractive error, nystagmus and strabismus were commonly associated with Sotos syndrome.[20]

Our patient had all the typical clinical features of Sotos syndrome. In addition, he had the rare association of primary optic atrophy and epilepsy. He had acromegalic features, normal endocrine function and a nonprogressive neurologic disorder after 13 years of age. No genetic study was performed in our patient. Although ocular manifestations have been described in Sotos syndrome, the presence of primary optic atrophy has been documented only in one patient.[7] Thus, Sotos syndrome, according to Srour *et al.*, is not uncommon and can be diagnosed among children with global developmental delay and macrocephaly.[12]

## References

[1] Sotos JF, Dodge PR, Muirhead D, Crawford JD, Talbot NB (1964). Cerebral gigantism in childhood. A syndrome of excessively rapid growth with acromegalic features and a non progressive neurologic disorder. N Engl J Med.

[2] Winship IM (1985). Sotos syndrome-autosomal dominant inheritance substantiated. Clin Genet.

[3] Hook EB, Reynolds JW (1967). Cerebral gigantism: Endocrinological and clinical observations of six patients including a congenital giant, concordant monozygotic twins and a child who achieved adult gigantic size. J Pediatr.

[4] Rio M, Clech L, Amiel J, Faivre L, Lyonnet S, Le Merrer M (2003). Spectrum of NSD1 mutations in Sotos and Weaver syndromes. J Med Genet.

[5] Cole TR, Hughes HE (1994). Sotos syndrome: A study of the diagnostic criteria and natural history. J Mol Genet.

[6] Yen MT, Gedde SJ, Flynn JT (2000). Unilateral glaucoma in sotos syndrome (cerebral gigantism. Am J Ophthalmol.

[7] Inoue K, Kato S, Numaga J, Sakurai M, Ohara C, Ouchi M (2000). Optic disk pallor and retinal atrophy in Sotos syndrome (cerebral gigantism). Am J Ophthalmol.

[8] Le Marec B, Pasquler L, Dugast C, Gesselin M, Odent S (1999). Gastric carcinoma in Sotos syndrome (cerebral gigantism). Ann Genet.

[9] Buyukgebiz A, Ercal D, Bober E (1996). Sotos syndrome with septo-optic dysplasia. J Pediatr Endocrinol Metab.

[10] Buyukgebiz A, Kinik E (1990). Sotos syndrome presenting with epilepsy. Turk J Pediatr.

[11] Di Marco G, Levantesi G, Parisi G, Chialrelli A (1989). Congenital cardiopathy in a patient with Sotos syndrome: Description of a case. G Ital Cardiol.

[12] Srour M (2006). Diagnosing Sotos syndrome in the setting of global developmental delay and macrocephaly. J Child Neurol.

[13] Douglas J, Hanks S, Temple IK, Davies S, Murray A, Upadhyaya M (2003). NSD1 mutations are the major cause of Sotos syndrome and occur in some cases of Weaver syndrome but are rare in other overgrowth phenotypes. Am J Hum Genet.

[14] Douglas J, Tatton-Brown K, Coleman K, Guerrero S, Berg J, Cole TR (2005). Partial NSD1 deletions cause 5% of Sotos syndrome and are readily identifiable by multiplex ligation dependent probe amplification. J Med Genet.

[15] Visser R, Hasegawa T, Niikawa N, Matsumoto N (2006). Analysis of the NSD 1 promoter region in patients with Sotos syndrome phenotype. J Hum Genet.

[16] Schaefer GB, Bodensteiner JB, Beuchler BA, Lin A, Cole TRP (1997). The neuroimaging findings in Sotos syndrome. Am J Med Genet.

[17] Horikoshi H, Kato Z, Masuno M, Asano T, Nagase T, Yamagishi Y (2006). Neuroradiologic findings in Sotos syndrome. J Child Neurol.

[18] al Rasheed AA, al-Jarallah AA, Salih MA, Kolawole T, al-Jarallah J (1999). Sotos syndrome(cerebral gigantism): A clinical and radiological study of 14 cases from Saudi Arabia. Ann Trop Paediatr.

[19] Ray M, Malhi P, Bhalla AK, Singhi PD (2003). Cerebral gigantism with West syndrome. Indian Pediatr.

[20] Maino DM, Kofman J, Flynn MF, Lai L (1994). Ocular manifestations of Sotos syndrome. J Am Optom Assoc.

